# B cell MHC haplotype affects follicular inclusion, germinal center participation and plasma cell differentiation in a mouse model of lupus

**DOI:** 10.3389/fimmu.2023.1258046

**Published:** 2023-11-28

**Authors:** Camilla Wibrand, Thomas R. Wittenborn, Lasse Frank Voss, Gudrun Winther, Lisbeth Jensen, Alexey Ferapontov, Sofie V. Fonager, Cecilia Fahlquist-Hagert, Søren E. Degn

**Affiliations:** ^1^ Laboratory for Lymphocyte Biology, Department of Biomedicine, Aarhus University, Aarhus, Denmark; ^2^ Department of Health Technology, Technical University of Denmark, Kongens Lyngby, Denmark; ^3^ Center for Cellular Signal Patterns (CellPAT), Aarhus University, Aarhus, Denmark; ^4^ Interdisciplinary Nanoscience Center (iNANO), Aarhus University, Aarhus, Denmark

**Keywords:** B cells, MHC, autoimmunity, autoantibodies, germinal centers, complement, systemic lupus erythematosus, plasma cells

## Abstract

**Introduction:**

MHC class II molecules are essential for appropriate immune responses against pathogens but are also implicated in pathological responses in autoimmune diseases and transplant rejection. Previous studies have shed light on the systemic contributions of MHC haplotypes to the development and severity of autoimmune diseases. In this study, we addressed the B cell intrinsic MHC haplotype impact on follicular inclusion, germinal center (GC) participation and plasma cell (PC) differentiation in the context of systemic lupus erythematosus (SLE).

**Methods:**

We leveraged the 564Igi mouse model which harbors a B cell receptor knock-in from an autoreactive B cell clone recognizing ribonuclear components, including double-stranded DNA (dsDNA). This model recapitulates the central hallmarks of the early stages of SLE. We compared 564Igi heterozygous offspring on either H2b/b, H2b/d, or H2d/d background.

**Results:**

This revealed significantly higher germinal center (GC) B cell levels in the spleens of H2b/b and H2b/d as compared to H2d/d (p<0.0001) mice. In agreement with this, anti-dsDNA-antibody levels were higher in H2b/b and H2b/d than in H2d/d (p<0.0001), with H2b/b also being higher compared to H2b/d (p<0.01). Specifically, these differences held true both for autoantibodies derived from the knock-in clone and from wild-type (WT) derived clones. In mixed chimeras where 564Igi H2b/b, H2b/d and H2d/d cells competed head-to-head in the same environment, we observed a significantly higher inclusion of H2b/b cells in GC and PC compartments relative to their representation in the B cell repertoire, compared to H2b/d and H2d/d cells. Furthermore, in mixed chimeras in which WT H2b/b and WT H2d/d cells competed for inclusion in GCs associated with an epitope spreading process, H2b/b cells participated to a greater extent and contributed more robustly to the PC compartment. Finally, immature WT H2b/b cells had a higher baseline of BCRs with an autoreactive idiotype and were subject to more stringent negative selection at the transitional stage.

**Discussion:**

Taken together, our findings demonstrate that B cell intrinsic MHC haplotype governs their capacity for participation in the autoreactive response at multiple levels: follicular inclusion, GC participation, and PC output. These findings pinpoint B cells as central contributors to precipitation of autoimmunity.

## 1 Introduction

The major histocompatibility complex (MHC) locus is well-established as the main genetic determinant of susceptibility to infections and autoimmune diseases (1, 2). In humans, the MHC locus is also termed the human leukocyte antigen (HLA) locus, and it is the most polymorphic locus in the human population. The equivalent locus in mice, for historical reasons, is termed the H2 locus. In wild mice, this locus is also highly polymorphic, but in inbred strains of mice, monomorphic haplotypes of MHC have been fixed on both alleles (3). Thus, whereas in man and wild mice, co-dominant expression from maternal and paternal genes contributes to MHC diversity, inbred mice have only a single set of MHC. The MHC locus can be divided into three main regions: Class I, Class II and Class III. The class I region harbors the classical and non-classical HLA-I-like genes, the class II region harbors the classical and non-classical HLA-II-like genes, and the class III region harbors genes encoding complement components and cytokines (1). In humans, there are three related classical HLA-I molecules: HLA-A, HLA-B, and HLA-C, encoded by each their gene, the product of which pairs with β_2_-microglobulin to allow presentation of peptides to cytotoxic T cells on the surface of all nucleated cells. Similarly, there are three pairs of classical class II alpha and beta chain genes, encoding HLA-DP, -DQ and -DR, which are expressed on the surface of professional antigen-presenting cells and function to present peptides to T-helper cells. The class I locus is mirrored in mice by H2-K, H-2D and H-2L, each encoding a classical class I molecule in combination with β_2_-microglobulin. A number of strains, including C57BL/6 mice, however, lack the H2-L allele. The class II locus is mirrored by I-A and I-E, which encode two isoforms of classical class II alpha and beta chain genes. In several commonly used inbred strains of mice, including C57BL/6, 129 and BXSB, the I-E alpha-chain is a pseudogene, leaving only a single functional isoform of MHC-II (4).

Because of the monomorphic nature of MHC in inbred mice, they have been an ideal model organism to study the role of variants of MHC in peptide antigen presentation and its connection with susceptibility to infection and autoimmune disease. Much effort has gone into understanding these aspects, however, whereas this has yielded great insight into the nature of responses driven globally by antigen-presenting cells (APCs), less is known about the isolated role of specific subsets of APCs. Among APC subsets, B cells are particularly interesting because they are subject to elaborate diversification and selection processes which depend on their capacity to present peptides to T cells, and because their antigen-presentation is intimately linked with their adaptive receptor specificity. Thus, B cells have a powerful capacity to focus the immune response to particular antigenic targets (5). This is especially relevant in the context of autoimmunity, where we, and others, have shown that they play a critical role in breaking tolerance (6–9). However, isolating B cell antigen presentation *in vivo* in the context of a physiological response has historically been challenging as contributions from other antigen-presenting subsets such as dendritic cells and macrophages could not be excluded. Here we leverage a well-characterized B cell receptor (BCR) knock-in model with specificity for nucleic acid autoantigens, the 564Igi model (10), whereby the autoimmune response is naturally linked to BCR-driven antigen presentation. We investigate the immune response in 564Igi mice on H2b vs. H2d background, and further examine the B cell intrinsic effects by generating mixed bone marrow chimeras. Our model overcomes previous limitations because it allows studying the competition between highly polyclonal populations of wild-type derived B cells with different MHC composition within the same environment. This enables deciphering the MHC-dependent B cell intrinsic differences in capacity to partake in and contribute to the autoimmune process.

## 2 Materials and methods

### 2.1 Mice

C57BL/6JRj mice were obtained from Janvier Labs and H2d congenic mice (B6.C-*H2^d^/*bByJ, JAX stock #000359) were obtained from Jackson Laboratories. 564Igi mice (B6.129S4(Cg)-*Igk^tm1(Igk564)Tik^ Igh^tm1(Igh564)Tik^/*J) (10) were kindly made available by Thereza Imanishi-Kari, Tufts University, and provided by Michael C. Carroll, Boston Children’s Hospital. 564Igi mice were crossed to H2d background. Offspring were genotyped using digital droplet polymerase chain reaction (ddPCR) for the 564Igi heavy and kappa light chain and two ddPCR assays for H2b vs. H2d. Mice were housed in the Animal Facility at the Department of Biomedicine, Aarhus University, Denmark, under specific pathogen-free (SPF) conditions in individually ventilated cages, on a 12-hour light/dark cycle with standard chow and water *ad libitum*. Both male and female mice were used in experiments.

### 2.2 ddPCR assays for 564Igi heavy and light chain

564Igi Heavy chain: A ddPCR assay was set up for 564Igi heavy chain using 564Igi Heavy probe (TGA CAT CTG AGG ACT CTG CGG T) with 5’-FAM and 3’-MGBEQ, 564Igi Heavy Forward primer (TCC AGC ACA GTC TAC ATG CAA) and 564Igi Heavy Reverse primer (CCC CCG ATC TTG CAC AGT AAT), using a HEX-probe based housekeeping assay (Bio-Rad 10042962).

564Igi Kappa chain: a ddPCR assay was set up for 564Igi Kappa chain using 564 Kappa probe (ACC CAC TCA CGT TCG GTG CTG) with 5’-HEX and 3’-MGBEQ, 564Igi Kappa Forward primer (AGG CTG AAG ATG CTG CCA TT), and 564Igi Kappa Reverse primer (TTC AGC TCC AGC TTG GTC C), using a FAM-probe based housekeeping assay (Bio-Rad 10042959).

Both reactions were run with the following conditions: 95°C 5 min, then 40 cycles of 94°C 30 s, 62°C 45 s, and 72°C 45 s, followed by 96°C 5 min, and 12°C hold.

### 2.3 ddPCR assays for H2b vs. H2d

Assay 1: a ddPCR assay for H2b vs. H2d was established based on JAX Protocol 18706, using primers 22205 (CAC AGG AGA GAG GAT GTT CTG) as forward primer and 22206 (AGG GTT GCA GAT CCA TAA TTG) as reverse primer, with MGB probes 21943 (ATG CGA TGC GTG ATG AAG) carrying a FAM (channel 1) for H2b detection and 21944 (AAG GAT GCG TGA TGA AGG AT) carrying a HEX (channel 2) for H2d detection.

Assay 2: a ddPCR assay for H2b vs. H2d was established based on JAX Protocol 21973, using primers 22202 (CCA AGA ACC TCC TCT CTC ATG T) as forward primer and 22203 (GCA GAT GTC CCA CCC TAT TC) as reverse primer, with MGB probes 21926 (ATG GCT GGG CAG AAG AGA G) carrying a HEX (channel 2) for H2b detection and 21928 (ATG GCT GGG CTG AAG AGA G) carrying a FAM (channel 1) for H2d detection.

Both reactions were run with the following conditions: 95°C 5 min, then 40 cycles of 94°C 30 s, 60°C 45 s, and 72°C 45 s, followed by 96°C 5 min, and 12°C hold.

### 2.4 Ethics statement

All animal experiments were conducted in accordance with the guidelines of the European Community and were approved by the Danish Animal Experiments Inspectorate (protocol numbers 2017-15-0201-01348 and 2017-15-0201-01319).

### 2.5 Bone marrow chimeras

Recipient mice were irradiated with 9 Gy (internal dosimetry) in a MultiRad 350 (Faxitron), with 350 kV, 11.4 mA, a Thoraeus filter [0.75 mm Tin (Sn), 0.25 mm Copper (Cu), and 1.5 mm Aluminium (Al)], and with a beam-distance of 37 cm, as described (11, 12). Irradiated recipients were kept on antibiotic water (either 1 mg sulfadiazine together with 0.2 mg trimethoprim per mL drinking water, or 0.25 mg amoxicillin per mL drinking water) to avoid opportunistic infections. On the following day, donor mice were anesthetized with continuous flow of 4% isoflurane and euthanized by cervical dislocation. Femora, fibulae/tibiae, ossa coxae and humeri were harvested, mechanically cleaned, and rinsed in FACS buffer (PBS, containing 2% heat-inactivated fetal calf serum (FCS), and 1 mM ethylenediaminetetraacetic acid (EDTA)). The bones were crushed in a mortar to release the bone marrow (BM) cells, and the cell extract was then passed through a 70 µm cell strainer. The donor BM cells were counted in a Cellometer K2 cell counter (Nexcelom) using Acridine orange (AO) and propidium iodide (PI) (ViaStain AOPI staining solution). Donor marrow was depleted of NK and T cells using biotinylated anti-NK1.1, TCRβ, CD3ε, CD4, and CD8α, followed by magnetic separation using streptavidin microbeads according to the manufacturer’s instructions (MojoSort, BioLegend). Cells from the desired combinations of mice were then mixed according to the proportions mentioned in the figure legends, pelleted by centrifugation (200 *g*, 10 min, 4°C) and resuspended to 1*10^8^ cells/mL. The donor cell mixtures were used to reconstitute the recipient mice by retroorbital injection of 200 µL (containing a total of 20*10^6^ cells) into each recipient mouse.

### 2.6 Tissue and blood collection

Mice were anesthetized with isoflurane (055226, ScanVet), decapitated, and the blood was collected in 1.5 ml tubes. Spleen, IngLN and MesLN were harvested, and the spleen was divided using surgical scissors. Tissues were either placed into ice-cold FACS buffer for flow cytometry analysis or prepared for histology. For flow cytometry, spleens, and lymph nodes in ice-cold FACS buffer were mechanically dissociated using pestles and samples were filtered through 70 µm cell strainers. Spleen samples were centrifuged at 200 *g* for 5 minutes at 4°C, lysed in RBC lysis buffer (155 mM NH_4_Cl, 12 mM NaHCO_3_, 0.1 mM EDTA), incubated at RT for 3 minutes, centrifuged, and finally resuspended in FACS buffer. For histology, spleen slices were placed directly into cryo-molds containing Tissue-Tek O.C.T. media (4583, Sakura Finetek) and frozen at -20°C. Collected blood samples were allowed to coagulate for 1 hr, then centrifuged at 3,000 *g* for 10 minutes at room temperature, the supernatant was collected, and centrifuged again at 20,000 *g* for 3 minutes at 4°C. Serum samples were stored at -80°C.

### 2.7 Flow cytometry

Twenty µL Fc-block (553142, BD) diluted 1:50 in PBS and 100 µL of each sample was added onto a 96-well plate then incubated for 5-10 minutes on ice. Antibodies and fixable viability dye (65-0865-14, ThermoFisher Scientific) were diluted 1/500 and 1/2,000, respectively, in FACS buffer. One hundred µL antibody mix was added to each sample well and incubated for 30 minutes on ice. The plate was centrifuged at 200 *g* for 5 minutes at 4°C, supernatant was removed, and cells were fixed for 30 minutes in PBS, 0.9% formaldehyde (F1635, Sigma-Aldrich) at RT. Following fixation, the plates were centrifuged at 200 *g* for 5 minutes at RT, the supernatant discarded, and the samples resuspended in FACS buffer. Flow cytometry evaluation was performed the following day using a 4-laser (405 nm, 488 nm, 561 nm, 640 nm) BD LSRFortessa analyzer with FACSDiva software version 8.0.2 (BD Biosciences). Data were analyzed in FlowJo version 10.8.1. The hybridoma producing 9D11 anti-idiotypic antibody reactive toward the 564Igi clone was kindly provided by Elisabeth Alicot, Boston Children’s Hospital, and the antibody was produced, purified, and labeled in-house with iFluor-647. All other antibodies were from commercial sources.

### 2.8 Anti-dsDNA measurements by time-resolved immunofluorometric assay (TRIFMA)

FluoroNunc Maxisorp 96-well plates were coated with 100 µg/mL salmon sperm dsDNA (AM9680, ThermoFisher Scientific) in PBS and incubated overnight at 4°C. Wells were blocked with 200 µL TBS containing 1% bovine serum albumin (BSA) (A4503, Sigma-Aldrich) for 1 hour at RT and washed thrice with TBS/Tw (TBS containing 0.05% v/v Tween-20 (8.17072.1000, Merck)). Samples, standards, and quality controls were diluted in TBS/Tw containing 5 mM EDTA and 0.1% w/v BSA, and subsequently added in duplicates. The plates were incubated at 37°C for 1 hour. Wells were washed thrice in TBS/Tw and incubated with biotinylated goat-anti-mouse Ig (1010-08, Southern Biotech), 1 µg/ml TBS/Tw, at 37°C for 1 hour. Wells were washed 3 times in TBS/Tw, and Eu^3+^-labeled streptavidin (1244-360, PerkinElmer) diluted 1:1,000 in TBS/Tw containing 25 µM EDTA was subsequently added to the wells and incubated at RT for 1 hour. Finally, the wells were washed thrice with TBS/Tw, and 200 µL enhancement buffer (AMPQ99800, Amplicon) was added. The plate was shaken for 5 minutes and counts were read in a time-resolved fluorometry plate reader (Victor X5, PerkinElmer).

### 2.9 Complement assays

FluoroNunc Maxisorp 96-well plates were coated with 100 µl of: 1 µg/ml mannan (purified in-house from *S. cerevisiae* and kindly provided by Steffen Thiel, Aarhus University) in coating buffer (50 mM carbonate, pH 9.6); 10 µg/ml acetylated bovine serum albumin (AcBSA) (Sigma B2518) in coating buffer; 1 µg/ml mouse IgG from serum (Sigma I5381) in coating buffer; or 20 µg/ml Zymosan (Sigma Z4250); and incubated overnight at 4°C. Wells were blocked with 200 µL TBS containing 1 mg/ml human serum albumin (HSA) for 1 hour at RT and washed thrice with TBS/Tw. Serum samples were diluted 1/100 and 1/200 (mannan), or 1/200 and 1/400 (AcBSA and mIgG) in assay buffer (10 mM HEPES, 145 mM NaCl, 2 mM CaCl_2_, 1 mM MgCl_2_, pH 7.4), or 1/40 and 1/80 (Zymosan) in alternative buffer (10 mM HEPES, 145 mM NaCl, 5 mM MgCl_2_, 10 mM ethyleneglycoltetraacetic acid (EGTA), pH 7.4). Dilutions and setup were performed on ice, and 100 µl was added to each well. A standard curve was included based on serial dilution of 8 H2b/b sera arbitrarily assigned the value 1,000 mU. Following incubation for 1.5 hrs at 37°C, the wells were washed thrice with TBS/Tw/Ca^2+^ (mannan, AcBSA, mIgG) or TBS/Tw (Zymosan). For mannan, AcBSA and mIgG coats, biotinylated anti-mouse C4 Ab (Cedarlane CL7504) was added at 0.5 μg/ml TBS/Tw/Ca^2+^. For Zymosan coat, anti-mouse C3 Ab (Cedarlane CL7503) was added at 0.5 µg/ml TBS/Tw/Ca^2+^. Wells were incubated 1.5 hrs at room temperature with anti-C4 or anti-C3 Ab. The wells were then washed thrice with TBS/Tw/Ca^2+^ (mannan, AcBSA, mIgG) or TBS/Tw (Zymosan), and europium-labeled streptavidin 0.1 μg/ml TBS/Tw containing 25 μM EDTA was added. After 1 h incubation and wash, again thrice with TBS/Tw/Ca^2+^, enhancement solution (PerkinElmer) was added, followed by reading of time-resolved fluorescence in a plate reader (Victor X5, PerkinElmer).

### 2.10 Immunofluorescence staining of spleen sections and quantification of GCs

Immunofluorescence staining of spleen sections was carried out according to a previous protocol (13). Briefly, a Cryostar NX70 Cryostat (Fisher Scientific) was used to cut 16 µm thick spleen sections which were mounted on SuperFrost+ glass slides (ThermoFisher Scientific) and air dried. Then, sections were rinsed in PBS, fixed in acetone by incubation for 10 minutes at RT, and then rehydrated in PBS. Antibodies, diluted in staining buffer (PBS, 2% v/v FBS, 0.1% w/v sodium azide), were added to the slides and incubated overnight. The following day, sections were washed thrice and mounted using Fluorescence Mounting Medium (S3023, Dako). The following antibodies were used: CD169-PE Clone 3D6.112 (1:500, 142404, BioLegend), IgD-AF488 (1:500, 405718, BioLegend), Ki67-eFlour660 Clone SolA15 (1:500, 50-5698-82, ThermoFisher Scientific). Images were obtained on an Olympus V120 Upright Widefield fluorescence slide scanner with a digital monochrome camera (Hamatsu ORCA, Flash4.0V2) and a 2/3” CCD camera. Images were processed using OlyVia 0.3.2. Full spleen sections were imaged, and all follicles and GCs were counted for quantification. The number of GCs and follicles were counted based on the following criteria: follicles were defined as an IgD-positive region of minimum 80 µm in diameter in close proximity to a marginal zone. GCs were defined as located within a follicle with a cluster of Ki67+ cells in the IgD exclusion zone. The quantification was carried out blinded to the mouse ID and H2 status. Channel intensities were uniformly adjusted for visual clarity in represented micrographs, but quantification was performed on raw images throughout.

### 2.11 Statistical analyses

**Figure 1:**
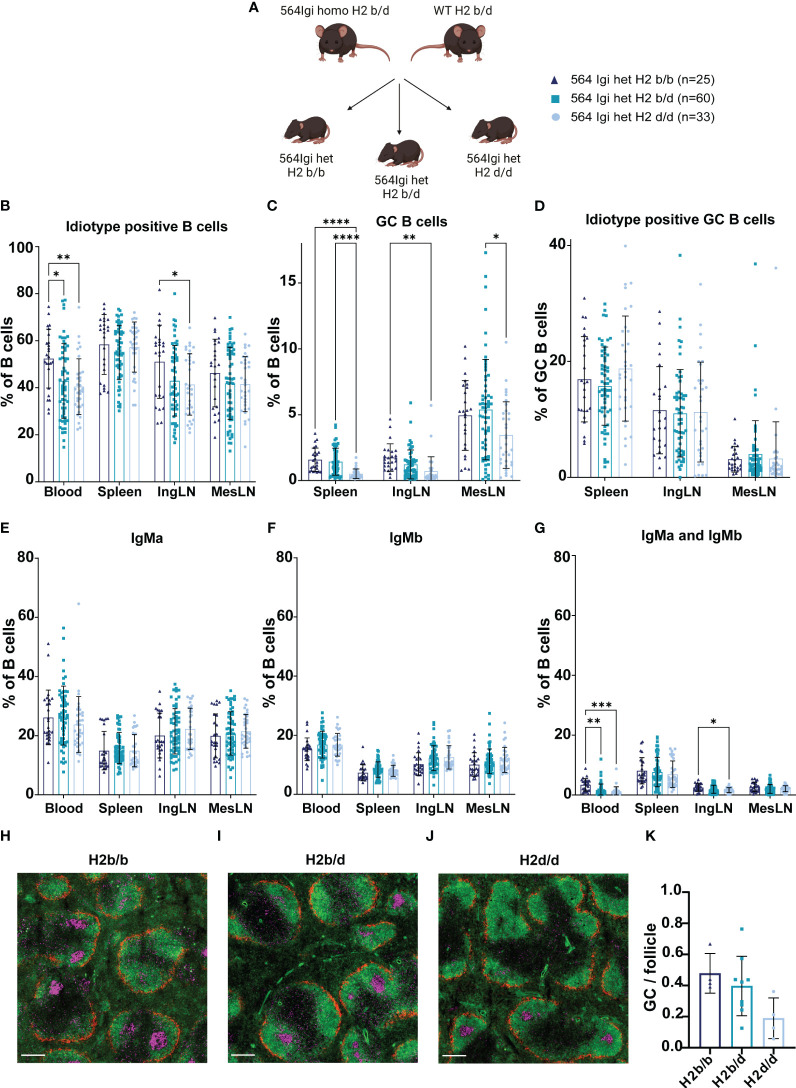
H2b/b background is more permissive for autoreactive B cell output and GC B cell formation than H2d/d. **(A)** Schematic example of experimental set-up. Graphical elements created in BioRender. **(B)** Frequency of idiotype positive B cells out of live, singlet B cells. **(C)** Frequency of GC B cells out of live, singlet B cells. **(D)** Frequency of idiotype positive GC B cells. **(E)** Frequency of IgMa positive cells out of live, singlet B cells. **(F)** Frequency of IgMb positive cells out of live, singlet B cells. **(G)** Frequency of IgMa and IgMb double positive cells out of live, singlet B cells. Data is pooled from 10 independent experiments. Each point represents one mouse. Bar graphs show mean ± SD. Data was analyzed using a mixed-effects analysis, matched by tissue, and Tukey’s post-test for multiple comparisons, comparing the mean of each column to all others. **(H)** Representative micrograph of an H2b/b spleen stained for marginal zone macrophages (CD169/MOMA-1, red), naïve follicular B cells (IgD, green) and dividing centrocytes (Ki67, purple). Scale bar indicates 500 µm. **(I)** Representative micrograph of an H2b/d spleen stained with same markers as panel **(H)** Scale bar indicates 800 µm. **(J)** Representative micrograph of an H2d/d spleen stained with same markers as panel **(H)** Scale bar indicates 800 µm. **(K)** Quantified frequency of GCs per follicle in the three haplotype groups. Each dot represents a mouse and bars show mean ± SD. Data was analyzed using a one-way ANOVA and Tukey’s post-test for multiple comparisons, comparing the mean of each column to all others. Micrographs were generated on a slide scanner by tile scanning and intensity adjusted for visual clarity. *p < 0.05, **p < 0.01, ***p < 0.001, ****p < 0.0001. Non-significant comparisons not shown.

**Figure 2:**
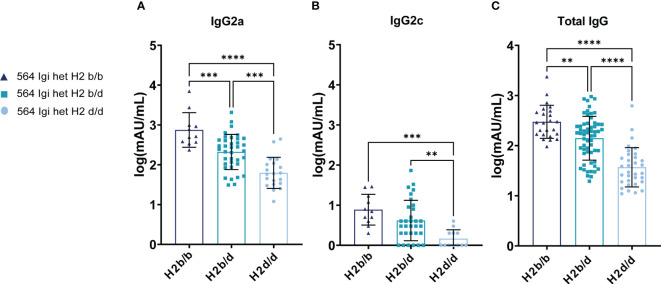
H2b/b background displays increased autoantibody production by the initiator clone and via epitope spreading compared to H2d/d. **(A)** Anti-dsDNA IgG2a antibody levels in 564Igi heterozygous mice on H2b/b (n=11), H2b/d (n=37), and H2d/d (n=20) background. **(B)** Anti-dsDNA IgG2c antibody levels of the same samples presented in panel **(A). (C)** Total anti-dsDNA IgG levels in 564Igi heterozygous mice on H2b/b (n=24), H2b/d (n=59), and H2d/d (n=33) background. Data is pooled from 9 independent experiments. Each point represents one mouse. All data points have been log transformed. Bar graphs show mean ± SD. Data was analyzed using an ordinary one-way ANOVA and Tukey’s post-test for multiple comparisons, comparing the mean of each column to all others. **p < 0.01, ***p < 0.001, ****p < 0.0001. Non-significant comparisons not shown.

**Figure 3:**
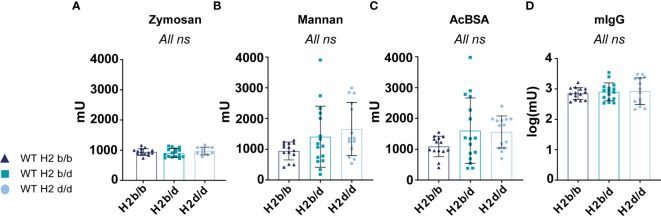
No marked differences in complement activity between H2 haplotype groups. Capacity for complement C3/C4 deposition via the alternative pathway activator zymosan **(A)**, lectin pathway MBL activator mannan **(B)**, lectin pathway ficolin activator AcBSA **(C)**, or classical pathway activator IgG **(D)**, of sera from WT H2b/b (n=14), H2b/d (n=16), and H2d/d (n=9 for zymosan, n=14 for other surfaces) mice. Data is pooled from 2 independent experiments. Each point represents one mouse. Bar graphs show mean ± SD. Data was analyzed using an ordinary one-way ANOVA and Tukey’s post-test for multiple comparisons, comparing the mean of each column to all others. ns = p ≥ 0.05, no significant difference.

**Figure 4:**
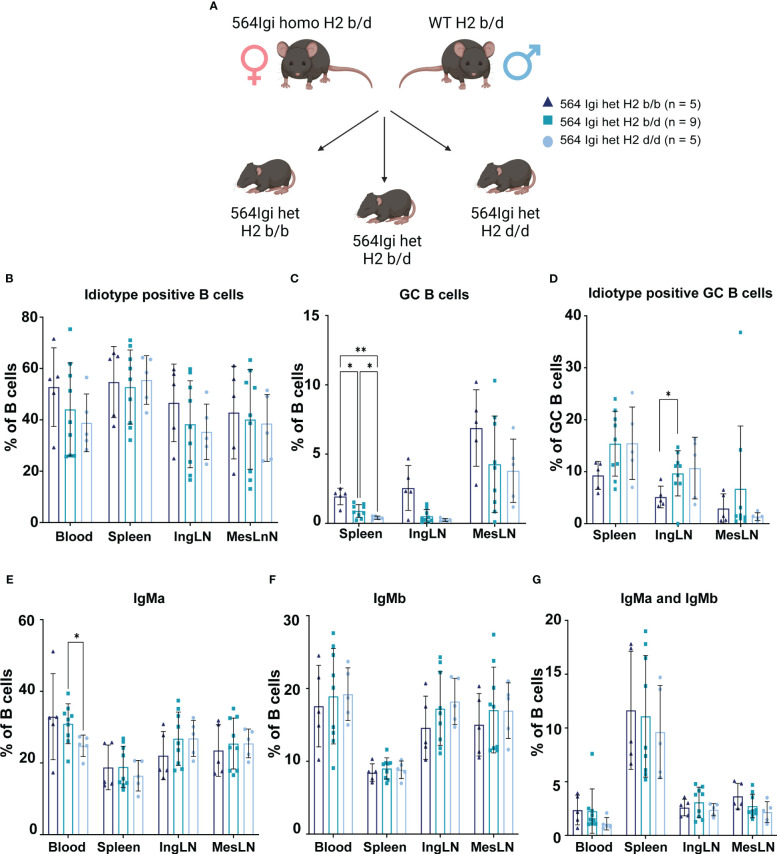
GC phenotype segregation with H2 haplotype is confirmed in littermates from single cross. **(A)** Schematic overview of experimental set-up. Graphical elements created in BioRender. **(B)** Frequency of idiotype positive B cells among live, singlet B cells. **(C)** Frequency of GC B cells out of live, singlet B cells. **(D)** Frequency of idiotype positive GC B cells. **(E)** Frequency of IgMa positive cells out of live, singlet B cells. **(F)** Frequency of IgMb positive cells out of live, singlet B cells. **(G)** Frequency of IgMa and IgMb double positive cells out of live, singlet B cells. Data is pooled from 2 experiments based on subsequent litters from the same cross. Each point represents one mouse. Bar graphs show mean ± SD. Data was analyzed using two-way ANOVA and mixed-effects analysis with Tukey’s post-test for multiple comparisons, comparing the mean of each column to all others. *p < 0.05, **p < 0.01. Non-significant comparisons not shown.

**Figure 5:**
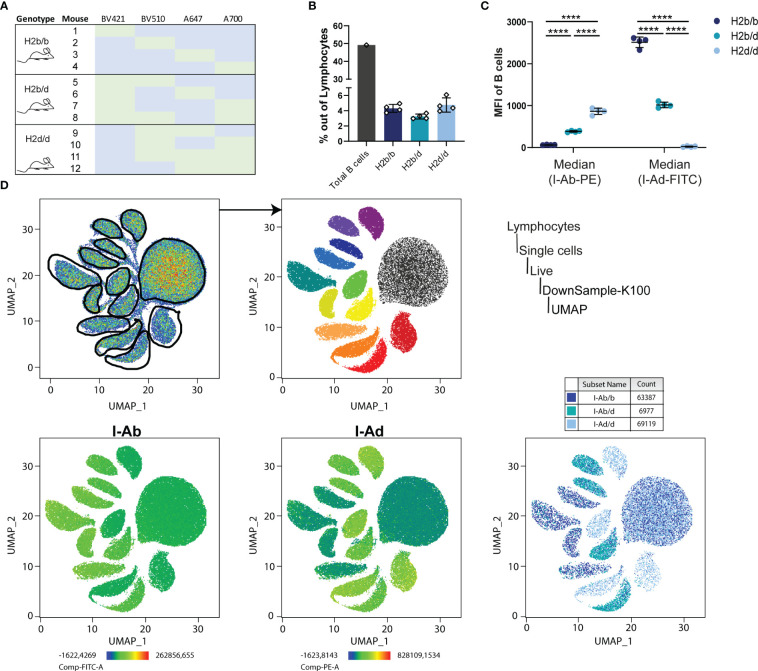
Expression of I-A on B cells of H2b/b, H2b/d and H2d/d mice. **(A)** To investigate the expression of the MHCII haplotypes in an unbiased manner, splenocytes purified from four mice of each genotype were barcoded with unique combinations of four fluorochrome-conjugated anti-B220 antibodies, as indicated in the table (green color indicates stained with that antibody and blue indicates no exposure). All the splenocytes were then combined in one tube and stained for I-A^b^, I-A^d^ and with a live/dead marker, followed by flow cytometry analysis. The sample was randomly downsampled to 100,000 cells prior to UMAP. **(B)** Total amount of B cells was measured as the sum of the cell count from all twelve samples together (grey bar) and specifically for each sample according to haplotype group (colored bars). Error bars represent the standard deviation of the mean for the 4 individual mice per group. **(C)** The median fluorescence intensity (MFI) was calculated out of all B cells for I-A^b^ and I-A^d^. Bars and error bars represent mean ± SD, n = 4 mice per group. Statistical significance given for two-way ANOVA with Tukey’s post-test, as ****p<0.0001. **(D)** A UMAP projection was generated based on the 4 index parameters, revealing 13 relatively discrete clusters, which were gated for downstream analyses (top row of panels). Expression of I-A^b^ and I-A^d^ was mapped onto the UMAP plot (bottom row of panels).

**Figure 6:**
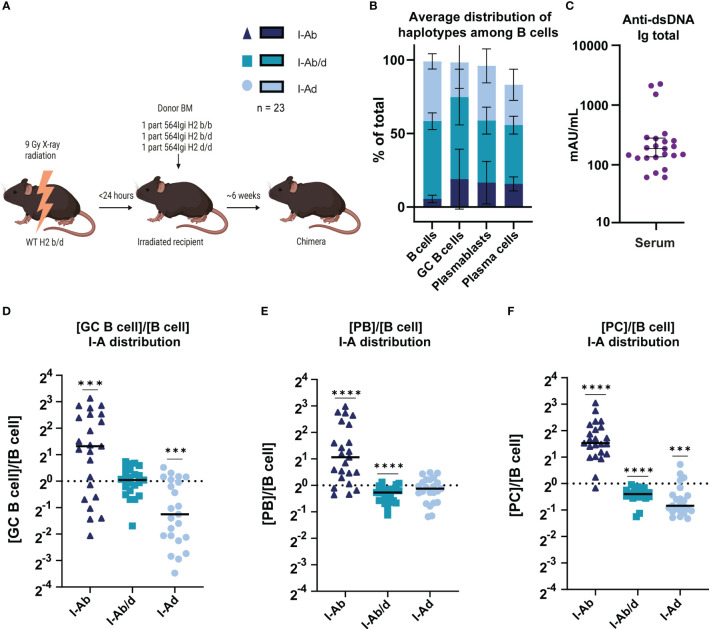
564Igi autoreactive knock-in BCR B cells on H2b background are more prone to contribute to germinal center, PB and PC populations. **(A)** Schematic overview of experimental set-up. Graphical elements created in BioRender. **(B)** Average distribution of haplotypes among B cells, GC B cells, plasmablasts, and plasmacells of mixed chimeras (n=23). **(C)** Total anti-dsDNA IgG levels in the mixed bone marrow chimeras. **(D)** Fractional representation of I-A haplotypes among GC B cells relative to total B cells. **(E)** Fractional representation of I-A haplotypes among PBs relative to total B cells. **(F)** Fractional representation of I-A haplotypes among PCs relative to total B cells. In C-F, each point represents one mouse and bars show the median. Data was analyzed using Wilcoxon signed-rank test comparing the median to the value 1 (2^0^). ***p < 0.001, ****p < 0.0001. Non-significant comparisons not shown.

**Figure 7:**
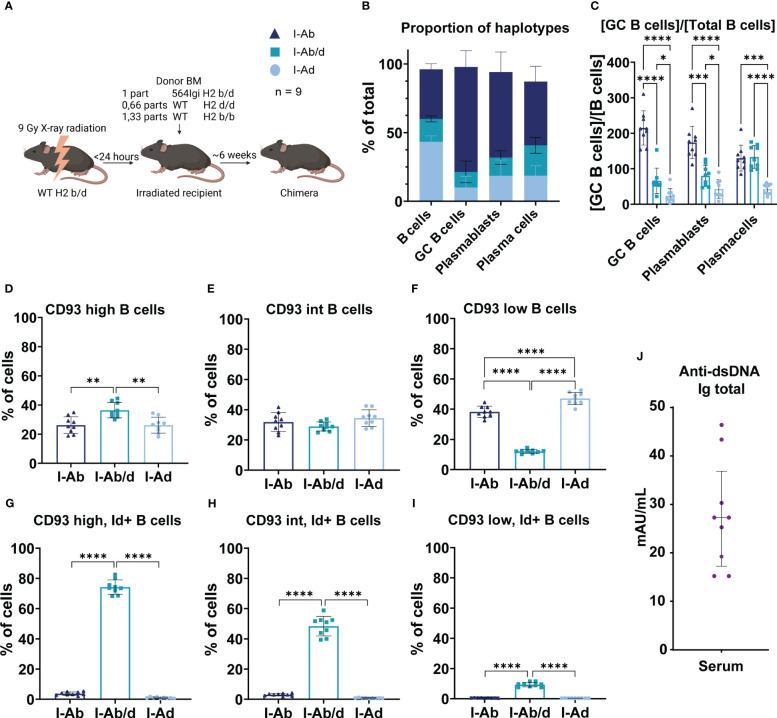
WT H2b B cells are more prone to participate in autoreactive epitope spreading, despite more stringent selection at the transitional stage. **(A)** Schematic overview of experimental set-up and legend explanation. Graphical elements created in BioRender. **(B)** Proportion of haplotypes across the compartments of interest. **(C)** Normalized haplotype frequency among GC B cells, plasmablasts and plasma cells relative to mature B cells. **(D)** Haplotype frequency among CD93 high B cells. **(E)** Haplotype frequency among CD93 intermediate (int) B cells. **(F)** Haplotype frequency among CD93 low B cells. **(G)** Haplotype frequency of CD93 high, idiotype positive B cells. **(H)** Haplotype frequency of CD93 int, idiotype positive B cells. **(I)** Haplotype frequency of CD93 low, idiotype positive B cells. **(J)** Total anti-dsDNA IgG levels in the mixed bone marrow chimeras. Each point represents one mouse. Bar graphs show mean ± SD. Data was analyzed using one- and two-way ANOVA with Tukey’s post-test for multiple comparisons, comparing the mean of each column to all others. *p < 0.05, **p < 0.01, ***p < 0.001, ****p < 0.0001. Non-significant comparisons not shown.

GraphPad Prism v. 9.4.0 was used for statistical analyses. Four tests for normality (D’Agostino-Pearson, Shapiro-Wilk, Anderson-Darling, and Kolmogorov-Smirnov) were performed for each data set. If two or more tests confirmed normality, it was accepted. If one test confirmed normality, QQ-plots were made to evaluate normality. Data that were neither normally distributed based on normality tests nor QQ-plots were log-transformed and re-tested for normality following the same procedure. Data in **Figure 1** were neither normally, nor log-normally distributed (IgMb was log-normally distributed but was not transformed to match IgMa). However, a non-parametric t-test for most datasets showed only minor changes in results (from e.g., * to **). Datasets with a change in the statistical result from statistically significant to non-significant or vice versa are illustrated in **Supplementary Table 1**. Data sets in **Figure 2** and mIgG in **Figure 3** were log-normally distributed, with the remainder of data in **Figure 3** being normally distributed. The entire datasets in **Figures 4**, **5** were normally distributed. In **Figure 6**, GC B cell + subgroups and plasmablast (PB) + subgroups were log-normally distributed, the rest being normally distributed. In **Figure 7**, 9D11-pos., H2-Kd and Kb were log-normally distributed (K-b/d was log-transformed as well), whereas the remainder was normally distributed. The nature of tests, n and meaning of bars, lines and error bars are indicated in the figure legends. All p values were adjusted for multiple comparisons, and significance is stated throughout as ns = p≥0.05, * = p<0.05, **= p<0.01, *** = p<0.001, and **** = p<0.0001.

## 3 Results

### 3.1 H2b/b background is more permissive for autoreactive B cell output and GC B cell formation than H2d/d

To investigate the B cell intrinsic role of MHC haplotype in the context of autoimmunity, we crossed the 564Igi autoreactive B cell receptor knock-in line (10), which was available on C57BL6/J (B6), i.e., H2b/b background, to the B6.C-*H2^d^/*bByJ line (B6.H2d). The B6.H2d line is in essence a B6 mouse, in which the H2d locus of the BALB/c strain has been introgressed, so the only locus that differs between regular B6 and B6.H2d is exactly the MHC locus. We established ddPCR assays for genotyping with copy-number determination of 564Igi heavy and light chains (Materials and Methods). We also established two ddPCR assays to monitor the H2 locus during the backcrossing of 564Igi heavy and light chains from H2b onto H2d background (Materials and Methods). Assay 1 targeted the 3’ UTR of H2-Q4 in the Qa locus of the murine H2 complex on Chromosome 17. Assay 2 targeted the 5’ end of H2-Eb2 in the I locus of the murine H2 complex on Chromosome 17. The two assays are spaced ~1 megabase apart, proximal to each their end of the H2 complex, providing a check on potential cross-over events during back-crossing of 564Igi heavy and light chains to H2d background (**Supplementary Figure 1A**). Representative data are shown in **Supplementary Figures 1B, C** for an H2b/b mouse (top left), H2b/d mouse (top right), H2d/d mouse (bottom left) and an H2 cross-over mouse (bottom right). Quantification is shown in **Supplementary Figure 1D**, where it can be seen that the representative H2b/b, H2b/d and H2d/d samples showed the expected ratio of H2b to H2d (100:0, 50:50, and 0:100, respectively), whereas the H2 cross-over mouse displayed an aberrant ratio (50:100) indicative of a cross-over event. During the introgression of the 564Igi heavy and light chains onto H2d background, we only observed this one cross-over event, which was excluded from subsequent crosses. We arrived at various crosses, in which either the sire or dam was 564Igi homozygous, and the other parent was WT, meaning that all offspring would be 564Igi heterozygous. Offspring was generated which could be H2d/d, H2b/d or H2b/b, or one of several combinations hereof, and we pooled offspring from multiple cohorts such that all three MHC haplotype combinations were robustly represented (**Figure 1A**), with a balanced female to male representation across groups (**Supplementary Table 2**). All offspring was genotyped using our two ddPCR assays for the H2 complex prior to inclusion in experiments.

Around 6-8 weeks of age we took blood and harvested spleens, inguinal lymph nodes (IngLN) and mesenteric lymph nodes (MesLN) for analysis by flow cytometry (see example gating strategy in **Supplementary Figure 2**). We determined the frequency of idiotype (9D11) positive cells, i.e., B cells that carried the knock-in receptor. In both heterozygous and homozygous 564Igi mice, all developing B cells initially express this receptor. However, due to homeostatic and central tolerance mechanisms, which work to prevent monoclonality of the B cell repertoire and autoreactivity, there is a significant physiological drive for secondary recombinations, V replacement and receptor editing (10, 11, 14). Still, in homozygous 564Igi mice, the idiotypic receptor makes up well over 90% of the circulating repertoire of B cells, as escape from the dual homozygous knock-in heavy and light chains is difficult. In heterozygous 564Igi mice, however, only about half of the circulating repertoire carries the idiotype on B6 background, as previously reported (11). In agreement with this, analysis of our cohorts revealed idiotype positive frequencies of around 50-60%, depending on tissue, in H2b/b offspring (**Figure 1B**). Of note, this frequency was lowered slightly in H2b/d and H2d/d offspring, across all tissues examined, although statistically significant differences were only observed in blood and IngLN (**Figure 1B**).

564Igi mice present with spontaneous autoreactive GCs (10, 11, 15). Using CD38^lo^CD95^hi^ gating to define GC B cells, we compared the magnitude of spontaneous GC responses between the three MHC haplotype combinations. This revealed a lowered GC B cell frequency in spleen and IngLN of H2d/d offspring compared to both H2b/b and H2b/d offspring or H2b/b offspring, respectively (**Figure 1C**). In the MesLN, H2d/d had significantly lower GC B cell levels than H2b/d. Of note, whereas spontaneous GCs in the IngLN and spleen are congruent with an autoimmune response driven by the autoreactive 564Igi knock-in receptor, spontaneous GCs are constitutively present in the MesLN in response to the gut microbiota, even in WT mice. In the 564Igi model, the response in MesLN likely represents a composite autoimmune and microbiota-directed response. This is substantiated by the observation that even in the absence of TLR7, which ablates autoreactive splenic and cutaneous lymph node GCs, GCs persist in the mesenteric lymph nodes (11, 15). In further agreement with this, evaluation of idiotype positivity among GC B cells across tissues indicated a higher frequency in spleen and IngLN, as compared to MesLN (**Figure 1D**). Of note, however, even in spleen and IngLN, the idiotype frequency was lower among GC B cells than among total B cells, likely a consequence of somatic hypermutation causing a loss of idiotype reactivity, as previously noted (11).

The constant region downstream of the 564Igi knock-in heavy chain V(D)J is of the Igha allotype, whereas the C57BL/6J-derived WT Igh locus of B6 and B6.H2d mice is of the Ighb allotype. Hence in heterozygous 564Igi offspring on either of the three H2 haplotype combination backgrounds, B cells that express the knock-in receptor also carry the linked Igha constant region, and B cells that escape the knock-in and express a WT receptor carry the linked Ighb constant region. For several of the Ig isotypes, these allotypes can be distinguished by allotype-specific antibodies. In the case of the mature repertoire of B cells characterized by expression of IgM, we can thus distinguish 564Igi B cells as IgMa vs. WT B cells as IgMb, within individual mice. Consequently, we asked whether there were any differences in IgMa vs. IgMb levels in mice of the three H2 haplotype combinations. However, we observed only very minor variations in IgMa and IgMb levels between the groups of offspring, with no systematic variation across tissues (**Figures 1E, F**). We also observed a low, but non-zero frequency of IgMa and IgMb double-positive B cells, which were significantly higher in blood of H2b/b, as compared to both H2b/d and H2d/d offspring, and in IngLN of H2b/b as compared to H2d/d offspring (**Figure 1G**). Generally, the process of allelic inclusion efficiently enforces exclusive expression of a single heavy chain (14), hence a more likely explanation for this observation is a higher level of immune complexes carried via complement receptor 2 (CR2) on the surface of non-cognate B cells (16) in H2b/b mice. Finally, we quantified GCs per follicle using imaging (**Figures 1H–K**), revealing a trend toward higher GC levels in H2b/b and H2b/d than H2d/d, although these differences did not reach statistical significance.

Taken together, our findings indicated that overall, the primary repertoire of B cells in 564Igi heterozygotes on either of the three H2 haplotype backgrounds was comparable. Yet H2b/b seemed to be more permissive for output of idiotype positive B cells, and H2d/d presented an attenuated spontaneous GC formation.

### 3.2 H2b/b background displays increased autoantibody production by the initiator clone and via epitope spreading compared to H2d/d

To unravel the potential functional implications of the observed decreased GC output in H2d/d mice compared to H2b/b and H2b/d groups, we analyzed the levels of autoantibodies in their serum. To this end, we again leveraged the linkage of knock-in vs. WT BCRs with Igha vs. Ighb respectively, which allowed us to discriminate the origin of the major Ig isotype involved in anti-dsDNA responses via IgG2a vs. IgG2c detection, respectively (11, 17, 18). Additionally, we included total anti-dsDNA IgG. This revealed that H2b/b background harbored the greatest level of 564Igi B cell-associated anti-dsDNA of the IgG2a allotype, with H2b/d intermediate, and H2d/d the lowest (**Figure 2A**). The same trend was apparent for WT-derived IgG2c, although the difference between H2b/b and H2b/d did not reach statistical significance (**Figure 2B**). Indeed, we have previously shown that 564Igi heterozygotes display epitope spreading among WT-derived clones (11), and found the same to be the case in H2b/b mixed chimeras (11) and mixed chimeras consisting of H2b/b with H2b/d and H2d/d (6). Finally, the total anti-dsDNA IgG levels were congruent with the iso-allotype pattern, with a statistically significant stepwise increase in autoantibody levels going from H2d/d to H2b/d and finally H2b/b background (**Figure 2C**).

These observations were overall congruent with the observed GC B cell differences presented in **Figure 1C**, suggesting that differential GC B cell engagement on the three backgrounds could explain differences in autoantibody levels.

### 3.3 No marked differences in complement activity between H2 haplotype groups

The H2 locus also harbors class III genes, which importantly include many complement genes (1). Complement is known to be a central contributor to lupus-like disease, with particularly defects in early classical complement pathway components predisposing to lupus (19). Furthermore, incorporation of complement fragments in immune complexes may significantly modulate B cell activation by cognate antigen (20). Therefore, we considered the possibility that the differences observed in our B cell intrinsic lupus model on different MHC backgrounds could be attributed to differences in complement activity. To determine this in the absence of any potential confounding secondary effects from altered disease phenotypes, we analyzed the baseline complement activity in B6 mice of H2b/b vs. H2b/d, vs. H2d/d background. Sera from mice of the different backgrounds were incubated on surfaces coated with the alternative pathway activator Zymosan, the lectin pathway activator through MBL, mannan, the lectin pathway activator through ficolins, acetylated BSA (AcBSA), or finally murine IgG, a classical pathway activator. Reading out C3 (alternative pathway) or C4 (classical/lectin pathway) deposition, no statistically significant differences were found between the three groups, indicating that they were overall similar in terms of complement activity, at least up until the C3 or C4 convertase, through the three activation pathways (**Figure 3**).

### 3.4 GC phenotype segregation with H2 haplotype is confirmed in littermates from single cross

Having substantiated that the differences observed between H2 haplotype backgrounds in the 564Igi model with regards to autoreactive B cell activation were likely not determined by differences in complement activity, but rather classical MHC genes, most likely MHCII, we sought to corroborate these differences further. One issue with our initial experiments was that the diverse breeding setups employed, although random, could potentially affect phenotypes in the offspring. For example, we have previously shown in another autoimmune model, that offspring can receive quite robust levels of autoantibodies from autoimmune mothers (18), and hence having 564Igi dams in breeding setups with WT sires could exacerbate the phenotype in offspring, compared to those with the reverse breeding scheme. This could further be compounded by differences in maternal Ig levels based on their H2 background. Additionally, differences in maternal microbiome depending on 564Igi status and H2 status could be envisioned to potentially exacerbate such influences on offspring.

Accordingly, we devised a single cross, in which we could generate H2b/b, H2b/d and H2d/d littermates with 564Igi heterozygous status (**Figure 4A**). We observed the same trend in terms of idiotype levels as in **Figure 1B**, although this did not reach statistical significance due to the more limited number of mice per group (**Figure 4B**). For GC B cell levels, the picture was also the same as for **Figure 1C**, again with less statistical power, but significant differences between H2b/b, H2b/d and H2d/d were observed in the spleen (**Figure 4C**). IgMa and IgMb levels were comparable between groups, as seen in the larger cohort study (**Figures 4D, E**).

Altogether, these findings corroborated our observations from the larger cohort study, in a setting where we could exclude differences in maternal phenotype-, microbiota-dependent or other transgenerational influences.

### 3.5 The expression level of MHCII on B cells is in direct relation to the MHC haplotype

MHC molecules are generally thought to be co-dominantly expressed, however, to verify this we sought an unbiased approach to determine the relative expression levels of I-A^b^ and I-A^d^ on the surface of B cells using flow cytometry. To avoid subtle differences in staining index between samples, we devised a hashing strategy, in which splenocytes purified from 12 mice, four of each genotype, were each stained with a unique index derived from a combination of four fluorochrome-conjugated antibodies targeting B220 (**Figure 5A**). Following indexing, all 12 samples were pooled, then stained in a ‘one-pot’ reaction with anti-I-A^b^-PE and anti-I-A^d^-FITC, ensuring identical staining conditions. We verified that overall frequencies of I-Ab/b, I-Ab/d and I-Ad/d B cells were comparable, and B cells from the samples were equally represented, with a cumulative B cell frequency of around 50% (**Figure 5B**). We determined the MFI of I-Ab and I-Ad within each of the B cell populations (**Figure 5C**) following unbiased gating on a UMAP projection based on the 4 index parameters (**Figure 5D**). Of 13 discrete clusters, the larger corresponded to all non-B cells from the 12 samples, negative for all index markers, whereas the remaining 12 corresponded to the B cell population from each of the 12 samples (**Figure 5D**). As expected, expression mapping of I-A^b^ and I-A^d^ revealed that 8 of the clusters expressed I-A^b^, 8 expressed I-A^d^, with 4 expressing both (**Figure 5D**). The MFI for I-A^b^ and I-A^d^ clearly showed that both exhibited a gene-dosage-dependent expression profile, hence confirming their co-dominant expression (**Figure 5C**).

### 3.6 Autoreactive H2b B cells have intrinsic higher capacity to contribute to GC, PB and PC compartments

To directly compare the capacity of autoreactive B cells carrying either or both of the haplotypes to partake in the autoreactive response, we devised a mixed bone marrow chimera setup, in which these cells would be competing head-to-head within the same environment. WT H2b/d recipients were lethally irradiated, then reconstituted with 564Igi heterozygous H2b/b, H2b/d and H2d/d bone marrow (**Figure 6A**). Donor marrow was depleted of T cells and NK cells, to prevent graft vs. graft or graft vs. host reactions. The WT H2b/d recipients harbor thymic stroma equally capable of supporting development of both H2b and H2d restricted T cells. Looking at the relative frequencies of the haplotypes within the B cell compartment, it was clear that there was a heavy skew in favor of I-Ab/d and I-Ad/d B cells (**Figure 6B**), a phenomenon which we established was unrelated to the autoreactive phenotype (6), yet there was a more balanced representation of H2b/b within GCB, PB, and PC compartments. All emergent B cells at the outset carry the autoreactive knock-in receptor meaning that they will have the same starting affinity for autoantigen. We measured total anti-dsDNA IgG titers and all chimeras displayed robust levels (**Figure 6C**). To read out the competitive ability of autoreactive B cells carrying the different H2 haplotypes to partake in the autoreactive response we determined the fraction of each haplotype in GC (**Figure 6D**), PB (**Figure 6E**), and PC compartments of the spleen (**Figure 6F**), relative to their representation within the total B cell pool. As can be seen, I-Ab/b B cells were significantly overrepresented in all three compartments, I-Ab/d were modestly but significantly underrepresented in PB and PC compartments, whereas I-Ad/d were underrepresented among GC B cells and PCs.

In conclusion, I-Ab/b B cells carrying an autoreactive B cell receptor had an intrinsic higher capacity to contribute to the GC, PB and PC compartments than its I-Ab/d and I-Ad/d counterparts.

### 3.7 WT H2b B cells are more prone to participate in autoreactive epitope spreading, despite more stringent selection at the transitional stage

We have previously shown that in mixed chimeras with 564Igi bone marrow, the WT compartment(s) displays epitope spreading (11), and found that this can be extended to an H2d compartment, provided an MHC bridge is present (6). To delineate the H2 haplotype-dependent B cell intrinsic differences in potential for GC participation and GC output during epitope spreading, we devised a mixed chimera setup, in which WT H2b/b and WT H2d/d B cells would compete side by side within the same autoreactive environment. WT H2b/d recipients were lethally irradiated, then reconstituted with 564Igi homozygous H2b/d bone marrow, WT H2b/b and WT H2d/d bone marrow (**Figure 7A**). Donor marrow was depleted of T cells and NK cells, to prevent graft vs. graft or graft vs. host reactions. The WT H2b/d recipients harbor thymic stroma equally capable of supporting development of both H2b and H2d restricted T cells, and the 564Igi H2b/d bone marrow is ‘locked in’ to the autoreactive fate by the homozygous knock-in of heavy and light chains and is capable of initiating an autoreactive response and communicating with both H2b and H2d restricted T cells. The H2b and H2d restricted T cells would in turn be equally able to support break-of-tolerance of wild-type derived autoreactive B cells from H2b and H2d, compartments, respectively. Given the propensity for dominance of H2d compartments in the mature repertoire observed in **Figure 6**, the chimeras were set up at a pre-determined ratio of reconstitution, to allow approximately equally sized H2b and H2d B cell compartments. This, along with normal frequencies of all major cell subsets (**Supplementary Figure 3A**), was confirmed by blood sampling at 6 weeks post reconstitution and flow cytometric analysis of I-A (MHCII) and H2-K (MHCI) haplotype status (**Supplementary Figure 3B**). We also confirmed that idiotype positive cells were heavily represented among H2b/d B cells, as expected from the homozygous knock-in of heavy and light chains, and that circulating H2b/b and H2d/d cells were invariably idiotype negative, and hence characterized by a true WT repertoire (**Supplementary Figure 3C**).

At 8 weeks post reconstitution, the bone marrow chimeras were euthanized, and spleens were harvested and analyzed. We first confirmed the expected haplotype distribution within the B cell compartment (**Figure 7B**). Next, we asked to what extent B cells derived from the 564Igi initiator compartment versus the two distinct WT compartments were represented among GC B cells, PBs and PCs, relative to their representation among the mature B cell repertoire. This revealed that H2b derived WT B cells were much more apt at participating in the spontaneous autoreactive GC response, compared to H2d derived WT B cells, and surprisingly, also to a higher extent than 564Igi-derived B cells (**Figure 7C**). The finding of more robust GC participation of WT H2b cells as compared to their H2d WT counterparts was in line with the higher GC response of 564Igi mice on H2b background, as compared to H2d background. The relative dominance of WT H2b cells in GCs over 564Igi cells may be explained as a consequence of an increased propensity of autoreactive 564Igi B cells carrying a pre-rearranged and already hypermutated, and hence high-affinity, B cell receptor to immediately differentiate to PBs and PCs. Indeed, initial receptor affinity has previously been established as the main determinant of extrafollicular PB/PC activation vs. GC entry (21), and we have previously observed that 564Igi-derived B cells in mixed chimeras are poorly represented within GCs (11). In line with these considerations, H2b cells were also overrepresented among PBs, likely the immediate proportional output of GCs, whereas 564Igi cells ‘caught up’ to H2b cells in the PC population (**Figure 7C**). H2d cells were invariably underrepresented among all these populations (**Figure 7C**).

To understand if there were any differences in the WT precursor repertoires, we used CD93 (AA4.1) staining to define progressive maturation stages of splenic B cells from transitional to mature, as CD93 high, intermediate, and low. Segregating these by H2 status, it was evident that whereas H2b and H2d cells were relatively equally represented across CD93hi, int and lo, 564Igi H2b/d cells were overrepresented among CD93hi and severely underrepresented among CD93lo cell subsets (**Figures 7D–F**). This picture can be rationalized in terms of the relatively high output of 564Igi B cells of H2b/d status from the bone marrow, despite central tolerance mechanisms, due to their pre-rearranged receptor, followed by follicular exclusion at the transitional stage, preventing their full maturation. Indeed, this mechanism and its dependence on competition with WT B cells for BAFF has been thoroughly characterized previously (22, 23), and has been shown to operate in the 564Igi model (24). There was a slight, but statistically significant overrepresentation of WT H2d B cells among the CD93lo cell subset, compared to WT H2b cells, despite their equal levels at the preceding stages, indicating a more stringent negative selection of transitional WT H2b B cells (**Figure 7F**). Looking at idiotype positive frequencies within H2 haplotype groups stratified by CD93 status, it was clear that the drop in representation of 564Igi H2b/d B cells was accompanied by a progressive loss of idiotype positive cells, corroborating the notion that the ‘locked in’ autoreactive B cells were being selected out at the transitional checkpoint (**Figures 7G–I**). Nonetheless, a relatively robust level of anti-dsDNA IgG remained in the chimeras (**Figure 7J**), underscoring the established autoreactive environment, and in agreement with the representation of these cells in the PC pool (**Figure 7C**). The WT H2b and H2d populations were by-and-large idiotype negative, as would be expected, although there was a small frequency of idiotype positive cells among CD93hi and int H2b-derived cells (**Figures 7G, H**), indicating a higher baseline of B cells carrying a 564Igi-similar idiotypic element from H2b background. These cells were lost at the CD93lo stage (**Figure 7I**), supporting a more stringent pruning of this repertoire as causative of the slightly lower representation of H2b cells among the CD93lo subset (**Figure 7F**). Nonetheless, due to the efficient elimination of these cells, rendering the idiotype positive population essentially zero among CD93lo mature B cells (**Figure 7I**), we consider it a reasonable assumption that there would be no difference in the starting point for potential autoreactivity among B cell populations derived from H2b or H2d precursors. Importantly, because of the highly diverse repertoire of B cells from either of these two WT compartments, and the size of these compartments, wild-type derived autoreactive B cells would thus be sampled from a diverse pool of cells, precluding BCR-mediated differences on a population scale. Thus, the observed B cell intrinsic difference in propensity to partake in the autoreactive response seems directly related to the H2 status, because differences in secreted cytokines and complement components (although the latter was already largely ruled out at this point, see **Figure 3**) could be definitively excluded, as the competing B cells experience exactly the same environment.

All in all, our findings demonstrated an intrinsic higher capacity of H2b B cells to enter or persist in autoreactive GCs and contribute to their output, compared to their H2d counterparts. Importantly, these findings were obtained within a B cell driven autoreactive environment, in which diverse WT-derived repertoires of H2b and H2d cells competed on equal terms, within the exact same environment *in vivo.*

## 4 Discussion

The MHCII locus is well-known to play a substantial role in the development of autoimmune disease in general and in SLE specifically. However, and though much work has been done to investigate this, the pathways linking MHCII haplotypes to autoimmune phenotypes remain not fully elucidated. To investigate the B cell intrinsic contribution to this complex phenomenon, we utilized a well-described mouse model of SLE, the 564Igi mouse, and investigated not only the role of different MHCII haplotypes in the model itself, but also in two bone marrow chimera set-ups derived from this model.

As outlined in the introduction, different H2 haplotypes express different MHCII molecules. The more diverse MHCII molecules, the broader a peptide repertoire and the more opportunities to engage in an immune response. Indeed, H2d/H2z MHC haplotype heterozygosity acts as one major predisposing genetic element for autoimmune disease resembling SLE in the F1 hybrid of NZB (H2d) and NZW (H2z) mice (25). Following this train of thought, one might at the outset have expected 564Igi H2b/d mice to present with the highest levels of GC B cells, PCs and autoantibodies. However, this was contradicted by our results as H2b/d and b/b had similar and higher levels of GC B cells and anti-dsDNA-Abs than H2d/d, with a trend toward b/b being even more permissive of autoimmunity (**Figures 1**, **2**, **4**). *A priori*, a possible explanation for this could be that the surface density of each MHCII molecule is reciprocally connected to the number of different MHCII molecules, meaning that the density of each MHCII-peptide-complex in a H2b/d mouse would be lower than in an H2b/b or d/d mouse. Indeed, this was previously suggested by the observation of similar MHCII expression levels on cortical epithelial cells across various strains (26). However, we could rule out a ‘global cap’ on the number of MHCII molecules on the surface of B cells based on the co-dominant expression observed in **Figure 5**.

In the (NZB x NZW)F_1_ setting referenced above, Nishimura et al. suggested that the exacerbation of autoimmunity could be caused by the formation of mixed haplotype A_α_^d^A_β_^z^ heterodimers at low levels, which due to their scarcity would not adequately tolerize autoreactive T cells in the thymus (25). Clearly, a similar phenomenon cannot play a major role in 564Igi H2b/d mice, because these did not display a worse phenotype than their 564Igi H2b/b counterparts. On the contrary, previous studies have found that the Eα-gene – which as mentioned previously is not expressed in the H2b haplotype – plays a protective role in the development of SLE in lupus-prone mice, and affects mortality rates and levels of anti-dsDNA-Abs (27–31). Specifically, both in BXSB mice (32) and in (NZB x BXSB)F1 hybrid female mice (27) Merino et al. found a picture consistent with the present study, where H2b/d and H2b/b had higher and similar levels of anti-dsDNA-Abs and H2d/d had significantly lower levels. In (NZB x BXSB)F1 hybrid male mice, however, H2d/d developed SLE as severe as H2b/b and H2b/d backgrounds, demonstrating that the Yaa gene abrogates the MHC effect on murine lupus in (NZB x BXSB)F1 hybrid mice (27). Conversely, insertion of a transgene encoding I-E alpha chain could suppress autoimmunity in BXSB mice (33), but this was likely expressed at much higher than endogenous levels, as also noted for heterodimers in A_β_^z^ transgenic (NZB x NZW.H2d)F_1_ animals (25). Taken together, this suggests that the H2b haplotype contributes susceptibility to murine SLE, while H2d is a relatively resistant haplotype, and H2b exhibits a dominant effect on autoimmune responses.

To determine whether autoantibody was preferentially made by I-E-negative B cells, Eisenberg and colleagues irradiated (B6/lpr.Igha x B6/lpr.I-E_α_^d^)F1 mice and reconstituted these with equal amounts of B6/lpr.Igha and B6/lpr.I-E_α_^d^ bone marrow (28). They subsequently screened autoantibodies in allotype-specific ELISAs able to discriminate the Igha allotype from the B6-endogenous Ighb allotype, showing that most autoantibody (84-97%) was produced by I-E negative B cells. This result demonstrated that a functional I-E molecule in lpr mice leads to generalized reduction in autoantibody levels through a direct effect on B cells, however, the molecular mechanism of this effect was not determined. Around the same time, another group independently reported that E_α_^d^ transgene-mediated protection paralleled the expression levels of E_α_ peptide presented by I-Ab molecules, but not of I-E molecules on B cells, in comparative analyses of several BXSB and (MRL x BXSB)F_1_ transgenic lines and bone marrow chimeras (29). Taken together, this prompted a model of autoimmunity prevention based on competition for antigen presentation, in which excessive generation of E_α_ peptides prevents activation of would-be autoreactive T and B cells, by virtue of their high affinity to the I-A molecules.

Thus, there is a preexisting mechanistic framework for understanding the observations in the present study, which we believe adds novel insights on the relative contributions in the context of GC and PB/PC responses that were not previously investigated. Specifically, most prior studies were performed in complex genetic models that could not truly isolate the contribution of B cells, and the dominant negative effect of I-E was largely observed in transgenic models with vastly unphysiological expression levels (28, 29, 33). Here, we used a simple genetic model for B cell-driven autoreactivity on an otherwise healthy background (C57BL/6J) with either H2b/b, H2b/d or H2d/d MHC configurations. The model does not display severe pathology until very late in life, thus enabling studies of the MHC-dependent autoreactive B cell biology in a normal physiological context. We recapitulate the finding of exacerbated autoimmunity on H2b as compared to H2d background (**Figures 1**, **2**, **4**). We did not evaluate potential differences in output of the idiotypic receptor containing B cells and total B cells within the bone marrow of the different haplotypes. Of note, the increased frequency of idiotype positive B cells on H2b background (**Figure 1**) could at the same time be a primary read-out of a worsened autoreactive environment and a potential contributor to downstream phenotypic differences such as increased GCs and anti-dsDNA autoantibodies. In fact, we specifically demonstrate that when competing head-to-head with their H2b/d and H2d/d counterparts, H2b/b cells carrying an autoreactive knock-in BCR contribute to a greater extent to GC B, PB and PC compartments (**Figure 6**). This is important because, although few contacts are in principle enough for T cell engagement (34), it suggests a selective advantage directly based on MHC. It has previously been argued that cognate TCR:peptide-MHC interaction is the sole determinant of GC B cell selection (35, 36), although more recently it was demonstrated that only GC entry, not recycling is governed by peptide-MHC density (37).

Compared to H2d/d, H2b/d are slightly more represented in GC B and PC compartments, whereas H2d/d is slightly more represented among PBs, potentially indicating a preference for extrafollicular responses, which could be either T-dependent or T-independent.

Finally, we also investigated the B cell intrinsic role of H2 in the context of selection at the transitional stage and subsequent epitope spreading. This was done in three-donor chimeras using 564Igi H2b/d driver cells, WT H2b/b, and WT H2d/d donors. Here, the 564Igi cells should be equally able to communicate with the two WT compartments, and preferential selection of one over the other would reflect differential ability to partake in the autoimmune response. The analyses revealed that H2b/b were again vastly superior to H2d/d in terms of ability to contribute to the GC compartment (**Figure 7**). Stratifying for CD93 expression level, with immature transitional B cells expressing the highest, and mature naïve follicular B cells the lowest, revealed a heavy negative selection of the 564Igi derived cells. That is, the H2b/d cells were overrepresented among CD93 high and underrepresented among CD93 low B cells. Gating out the idiotype positive subset revealed a concomitant marked decrease of these cells from CD93 high to low. Among the WT compartments, H2b/b and H2d/d behaved overall similar, although H2d/d contributed significantly more to the mature CD93 low compartment than H2b/b. There was also a slightly higher idiotype positive background among the CD93 high and intermediate H2b/b cells, suggesting that the primary H2b/b repertoire might have a higher frequency of idiotypic B cells, which are then selected out during the transitional stage, resulting in a similar starting point in terms of repertoire of the mature compartment. Altogether, this demonstrated that even among B cells with a diverse B cell repertoire, H2b/b cells still display an increased propensity for partaking in autoreactive GC responses, compared to H2d/d.

Beyond MHCII molecules, additional factors associated with the MHC locus are known to play a role in autoimmunity, such as numerous complement factors (38) and several important cytokines including TNF-α (39). We found that functionally, complement levels did not differ significantly between the MHCII haplotypes up until the formation of the C3 convertase (**Figure 3**). More importantly, we could exclude contributions of such ‘environmental’ and soluble factors in the bone marrow chimera setups, where B cells carrying the 564Igi knock-in receptor (**Figure 6**) or a wild-type repertoire (**Figure 7**) were competing within the same environment.

To summarize, while previous studies have confirmed the importance of MHCII in autoimmunity generally, our study underlines the vital role of the MHCII haplotype in B cells in autoimmunity and show that the MHCII haplotypes themselves are intrinsically different in their autoimmune potential. This is of immense significance, as B cells are key players in any autoimmune response, and specifically in diseases such as SLE driven largely by autoantibody production and thereby differentiation of B cells. Dissecting the molecular pathways for this effect thus provides necessary steppingstones in the direction of understanding the pathology and etiology of autoimmune diseases.

## Data availability statement

The raw data supporting the conclusions of this article will be made available by the authors, without undue reservation.

## Ethics statement

The animal study was approved by The Danish Animal Experiments Inspectorate. The study was conducted in accordance with the local legislation and institutional requirements.

## Author contributions

CW: Formal Analysis, Writing – original draft, Writing – review & editing, Data curation, Investigation, Methodology, Visualization. TW: Data curation, Investigation, Methodology, Writing – review & editing, Supervision. LV: Data curation, Investigation, Methodology, Writing – review & editing. GW: Data curation, Investigation, Methodology, Writing – review & editing. LJ: Investigation, Methodology, Writing – review & editing. AF: Investigation, Methodology, Writing – review & editing. SF: Investigation, Methodology, Writing – review & editing. CF-H: Investigation, Methodology, Writing – review & editing, Data curation, Visualization. SD: Writing – review & editing, Conceptualization, Formal Analysis, Funding acquisition, Project administration, Resources, Supervision, Writing – original draft.
