# Supplementary material for: B cell MHC haplotype affects follicular inclusion, germinal center participation and plasma cell differentiation in a mouse model of lupus
Source: Front Immunol. 2023 Nov 28;14:1258046. doi: 10.3389/fimmu.2023.1258046 (PMC10715410; doi:10.3389/fimmu.2023.1258046)
Supplement: Supplementary Figure 1 [file DataSheet_1.docx]

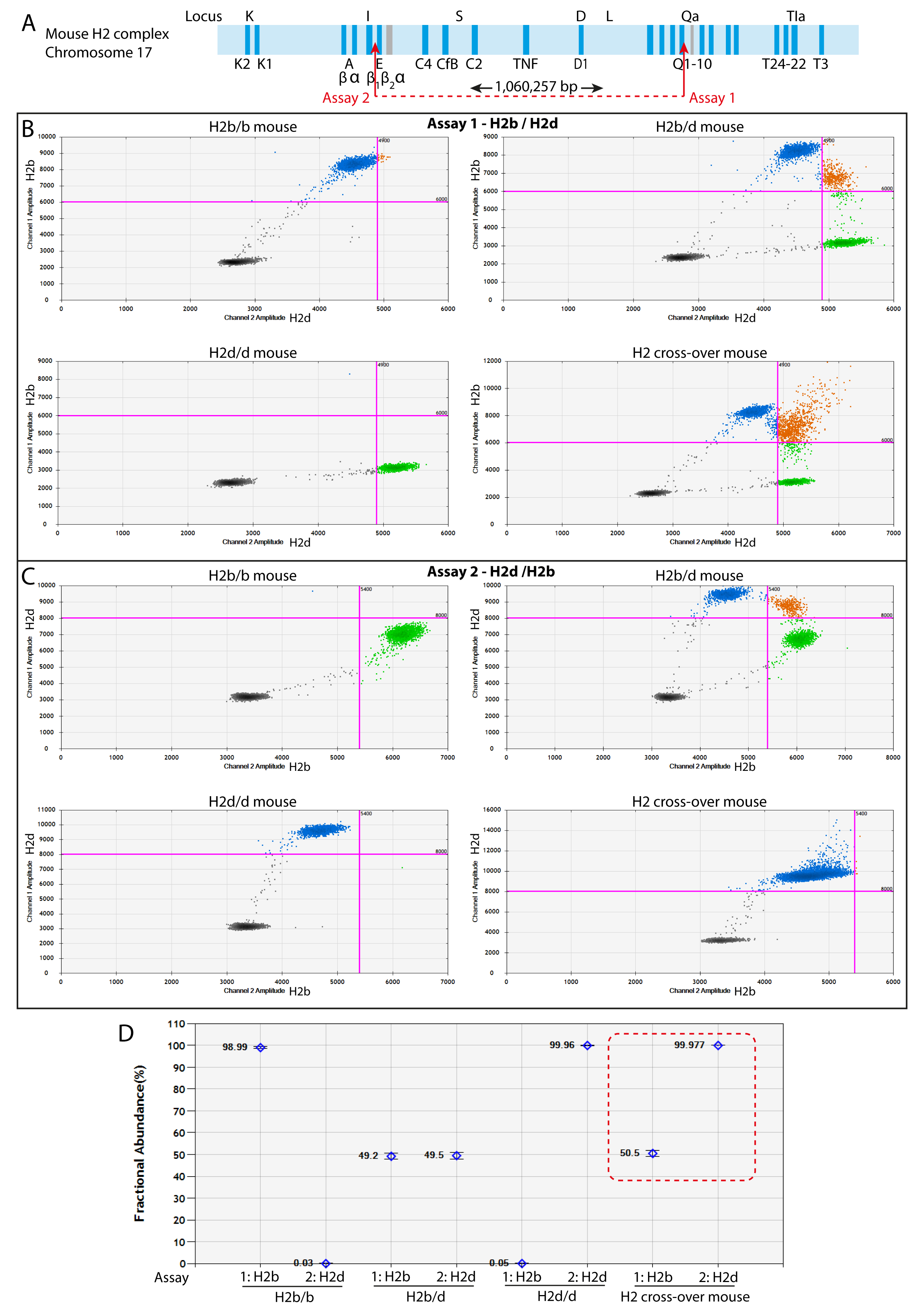


**SUPPLEMENTARY FIGURE 1. Overview of the H2 complex and ddPCR assays for H2 haplotype assignment with representative results.** (**A**) Heavily simplified schematic overview of the murine H2 complex on Chromosome 17 for the C57BL/6J strain (based on GRCm39, NCBI Reference Sequence NC_000083.7), with indication of ddPCR Assay 1 and Assay 2 employed in this study for haplotype assignment. As can be seen, Assay 1 targets the 3’ end of H2-Q4 of the Qa locus towards one end of the H2 complex, whereas Assay 2 targets the 5’ end of H2-Eb2 of the I locus towards the other end of the H2 complex. The two assays are spaced ~1 megabase apart. Important Class I (H2-K, -D and -L loci), Class II (I locus) and Class III (S locus) elements are indicated. Note that the I-E alpha chain is a pseudogene in C57BL/6J, as is Q5, and H-2L is missing from this strain, as is Q8. The schematic and the represented elements are not to scale. (**B**) Representative results for Assay 1 for an H2b/b mouse (upper left), an H2b/d mouse (upper right), an H2d/d mouse (lower left), and an H2 cross-over mouse (lower right), which in this assay looks like an H2b/d mouse. (**C**) Representative results for Assay 2 for an H2b/b mouse (upper left), an H2b/d mouse (upper right), an H2d/d mouse (lower left), and an H2 cross-over mouse (lower right), which in this assay looks like an H2d/d mouse. (**D**) Quantification of the representative results of Assay 1 and Assay 2, which shows agreement between Assay 1 and 2 for H2b/b (100:0), H2b/d (50:50) and H2d/d (0:100) mice, but disagreement for the H2 cross-over mouse with an aberrant 50:100 ratio of H2b to H2d alleles. This indicates that the H2 cross-over mouse carries one intact H2d allele and one allele in which a cross-over has occurred somewhere between the loci targeted by Assay 1 and Assay 2, such that the I-E locus (Assay 2) is from H2d, whereas the Qa locus (Assay 1) is from H2b.
