# Supplementary material for: B cell MHC haplotype affects follicular inclusion, germinal center participation and plasma cell differentiation in a mouse model of lupus
Source: Front Immunol. 2023 Nov 28;14:1258046. doi: 10.3389/fimmu.2023.1258046 (PMC10715410; doi:10.3389/fimmu.2023.1258046)
Supplement: Supplementary Figure 2 [file DataSheet_2.docx]

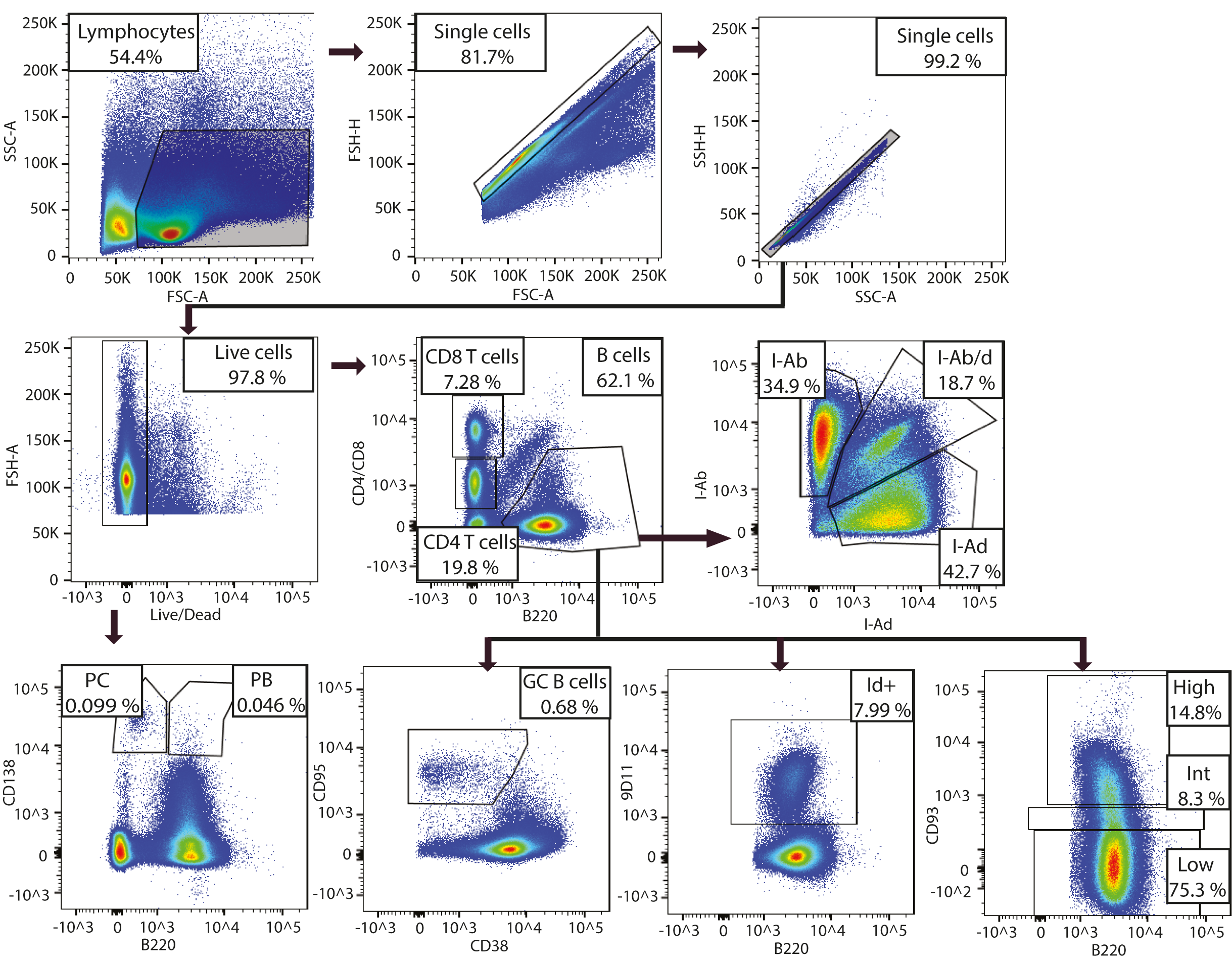


**SUPPLEMENTARY FIGURE 2. Overview of gating strategy based on a representative bone marrow chimera.** Lymphocytes were gated based on FSC-A vs. SSC-A, followed by singlet exclusion by SSC-H vs. SSC-A and FSC-H vs. FSC-A, and then live cells were gated by viability dye exclusion. CD4 and CD8 positive T cells were gated out, and similarly B cells were gated out as B220 positive. From the B cell gate, we determined 9D11 positive B cells, I-Ab, I-Ad and I-Ab+I-Ad positive B cells. Germinal Center B (GC B) cells were defined as CD38loCD95hi. Lastly, B cells were divided into three groups based on CD93 expression. Plasmablasts and plasma cells were gated out from live cells based on B220 and CD138 expression. In some experiments, IgMa, IgMb and IgMa+IgMb positive B cells were also gated (not shown). For gating of 9D11 positive cells in other populations than B cells, the same gate was used as that from B cells.
