# Supplementary material for: B cell MHC haplotype affects follicular inclusion, germinal center participation and plasma cell differentiation in a mouse model of lupus
Source: Front Immunol. 2023 Nov 28;14:1258046. doi: 10.3389/fimmu.2023.1258046 (PMC10715410; doi:10.3389/fimmu.2023.1258046)
Supplement: Supplementary Figure 3 [file DataSheet_3.docx]

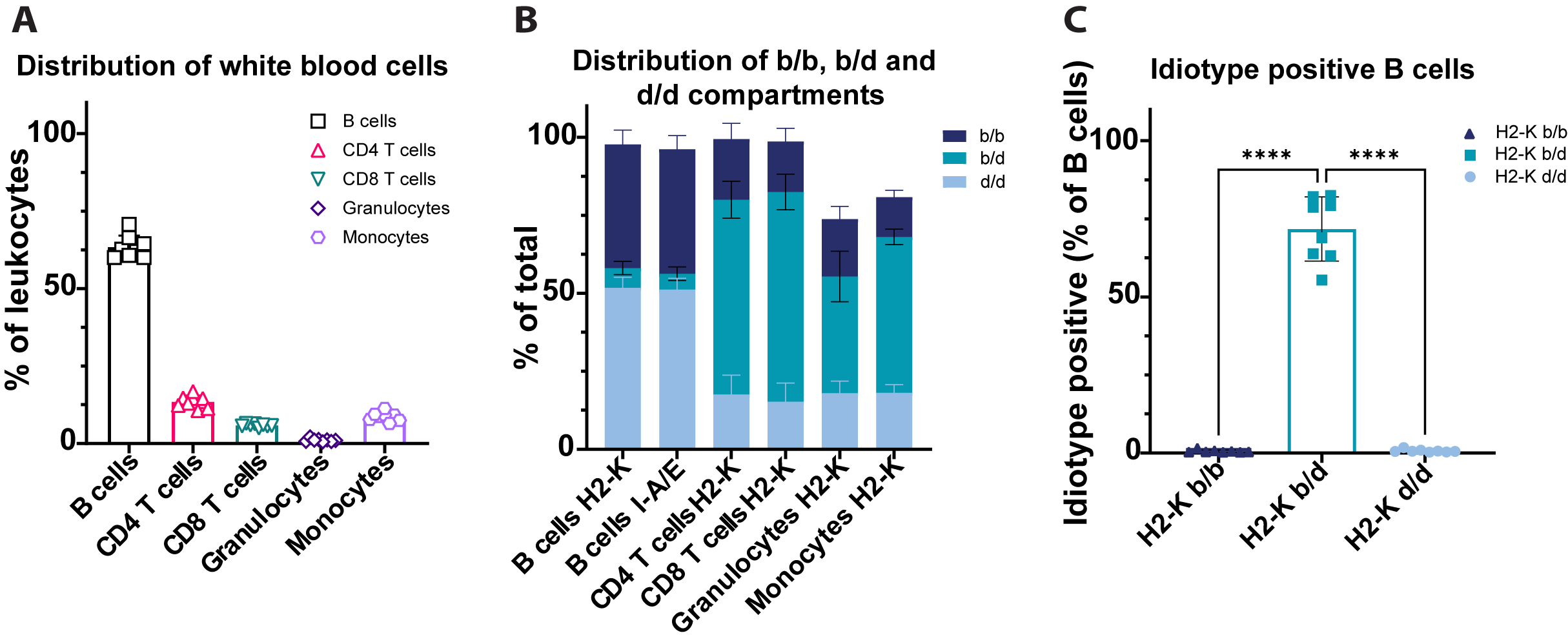


**SUPPLEMENTARY FIGURE 3. Supporting data for main Figure 7 based on FACS typing of chimeras.** (**A**) Distribution of white blood cells. (**B**) Distribution of b/b, b/d and d/d compartments. (**C**) Idiotype positive B cells. Bar graphs show mean ± SD. Data was analyzed using a one-way ANOVA with Tukey’s post-test for multiple comparisons, comparing the mean of each column to all others. **** = p < 0.0001. Non-significant comparisons not shown.
