# Supplementary material for: B cell MHC haplotype affects follicular inclusion, germinal center participation and plasma cell differentiation in a mouse model of lupus
Source: Front Immunol. 2023 Nov 28;14:1258046. doi: 10.3389/fimmu.2023.1258046 (PMC10715410; doi:10.3389/fimmu.2023.1258046)
Supplement: Supplementary file 4 [file Table_1.docx]

**Supplementary Table 1.** Overview of datasets for which non-parametric t-test gave divergent result.

| Tissue | Cell type | Group (low) | Group (high) | Change |
| --- | --- | --- | --- | --- |
| Blood | Haplotype positive B cells | H2 b/d | H2 b/b | ns to * |
| Inguinal LN | B cells | H2 d/d | H2 b/b | ns to * |
| Inguinal LN | GC B cells | H2 d/d | H2 b/d | ns to *** |
| Inguinal LN | GC B cells | H2 b/d | H2 b/b | ns to * |
| Mesenteric LN | B cells | H2 d/d | H2 b/d | * to ns |
| Mesenteric LN | CD4 T cells | H2 d/d | H2 b/b | * to ns |
