# Supplementary material for: B cell MHC haplotype affects follicular inclusion, germinal center participation and plasma cell differentiation in a mouse model of lupus
Source: Front Immunol. 2023 Nov 28;14:1258046. doi: 10.3389/fimmu.2023.1258046 (PMC10715410; doi:10.3389/fimmu.2023.1258046)
Supplement: Supplementary file 5 [file Table_2.docx]

**Supplementary Table 2.** Overview of sex distribution for 564het cohort animals.

|  | H2b/b | H2b/d | H2d/d | Total |
| --- | --- | --- | --- | --- |
| Female | 13 | 28 | 13 | 54 |
| Male | 12 | 32 | 20 | 64 |
| Total | 25 | 60 | 33 | 118 |
